# Nanoparticulate flurbiprofen reduces amyloid-β_42_ generation in an *in vitro* blood–brain barrier model

**DOI:** 10.1186/alzrt225

**Published:** 2013-11-27

**Authors:** Sabrina Meister, Iavor Zlatev, Julia Stab, Dominic Docter, Sandra Baches, Roland H Stauber, Mordechai Deutsch, Reinhold Schmidt, Stefan Ropele, Manfred Windisch, Klaus Langer, Sylvia Wagner, Hagen von Briesen, Sascha Weggen, Claus U Pietrzik

**Affiliations:** 1 Institute of Pathobiochemistry, University Medical Center of the Johannes Gutenberg University Mainz, Mainz, Germany; 2 Institute of Pharmaceutical Technology and Biopharmacy, University of Muenster, Muenster, Germany; 3 Department of Cell Biology and Applied Virology, Fraunhofer Institute for Biomedical Engineering, St. Ingbert, Germany; 4 Molecular and Cellular Oncology/Mainz Screening Center (MSC), ENT-Department, University Medical Center of the Johannes Gutenberg University Mainz, Mainz, Germany; 5 Department of Neuropathology, Heinrich Heine University, Duesseldorf, Germany; 6 The Biophysical Interdisciplinary Schottenstein Center for the Research and Technology of the Cellome, Bar Ilan University, Ramat gan, Israel; 7 Department of Neurology, Medical University of Graz, Graz, Austria; 8 NeuroScios GmbH, St. Radegund/Graz, Austria

## Abstract

**Introduction:**

The amyloid-β_42_ (Aβ_42_) peptide plays a crucial role in the pathogenesis of Alzheimer’s disease (AD), the most common neurodegenerative disorder affecting the elderly. Over the past years, several approaches and compounds developed for the treatment of AD have failed in clinical studies, likely in part due to their low penetration of the blood–brain barrier (BBB). Since nanotechnology-based strategies offer new possibilities for the delivery of drugs to the brain, this technique is studied intensively for the treatment of AD and other neurological disorders.

**Methods:**

The Aβ_42_ lowering drug flurbiprofen was embedded in polylactide (PLA) nanoparticles by emulsification-diffusion technique and their potential as drug carriers in an *in vitro* BBB model was examined. First, the cytotoxic potential of the PLA-flurbiprofen nanoparticles on endothelial cells and the cellular binding and uptake by endothelial cells was studied. Furthermore, the biological activity of the nanoparticulate flurbiprofen on γ-secretase modulation as well as its *in vitro* release was examined. Furthermore, the protein corona of the nanoparticles was studied as well as their ability to transport flurbiprofen across an *in vitro* BBB model.

**Results:**

PLA-flurbiprofen nanoparticles were endocytosed by endothelial cells and neither affected the vitality nor barrier function of the endothelial cell monolayer. The exposure of the PLA-flurbiprofen nanoparticles to human plasma occurred in a rapid protein corona formation, resulting in their decoration with bioactive proteins, including apolipoprotein E. Furthermore, luminally administered PLA-flurbiprofen nanoparticles in contrast to free flurbiprofen were able to modulate γ-secretase activity by selectively decreasing Aβ_42_ levels in the abluminal compartment of the BBB model.

**Conclusions:**

In this study, we were able to show that flurbiprofen can be transported by PLA nanoparticles across an *in vitro* BBB model and most importantly, the transported flurbiprofen modulated γ-secretase activity by selectively decreasing Aβ_42_ levels. These results demonstrate that the modification of drugs via embedding in nanoparticles is a promising tool to facilitate drug delivery to the brain, which enables future development for the treatment of neurodegenerative disorders like AD.

## Introduction

Alzheimer’s disease (AD) is an age-related neurodegenerative disorder currently affecting more than 35 million people worldwide [1]. To date, the treatment of AD is only symptomatic and there is no cure for the disease [2]. AD is characterized by neuronal and synaptic loss, neurofibrillary tangle formation and extracellular deposits of amyloid-β (Aβ) peptides in susceptible brain regions, which result in learning and memory impairment [3]. Aβ is generated through the sequential processing of the amyloid precursor protein (APP) by the β-secretase (BACE1) and the γ-secretase complex, and occurs in various isoforms between 36 and 46 amino acids in length, with Aβ_40_ and Aβ_42_ being the most prevalent variants [4-6]. Recently, we have demonstrated that APP is also processed by the metalloprotease meprin β, which might act as an additional enzyme, responsible for the release of N-terminal truncated Aβ species and soluble N-terminal APP fragments, independent of BACE1 [7,8]. According to the amyloid hypothesis [9-11], abnormal accumulation or increased generation of Aβ_42_ peptides in the brain is a primary event in the pathogenesis of AD [12-14]. Therefore, several strategies such as reducing Aβ generation, blocking its aggregation or enhancing Aβ clearance in the brain are thought to slow down the progression of the disease [15].

Besides APP, γ-secretase has more than 50 substrates with critical functions, such as cell signaling (*for example,* the Notch receptor), cell adhesion and apoptosis [16]. In earlier studies, we were able to demonstrate that the treatment of Chinese hamster ovary (CHO) cells with some nonsteroidal anti-inflammatory drugs (NSAIDs) such as indomethacin, ibuprofen and flurbiprofen specifically decreased the secretion of the Aβ_42_ peptides. This was accompanied by an increase of other Aβ isoforms (for example, Aβ_37_ and Aβ_38_), indicating that NSAIDs subtly altered γ-secretase activity without significant impairment of other APP processing pathways or Notch signaling [17]. NSAIDs exert their principal therapeutic effects, reducing fever, pain and inflammation, by blocking the cyclooxygenase (COX)-mediated synthesis of inflammatory prostaglandins [18]. However, some NSAIDs were shown to selectively lower Aβ_42_ production *in vitro* and in mouse models of AD, independently of COX activity [17,19]. Later, small molecules with the ability to lower Aβ_42_ production without altering overall γ-secretase activity were termed γ-secretase modulators (GSM) [20]. Recently, the clinical development of the Aβ_42_ lowering agent tarenflurbil, the COX-inactive *R*-enantiomer of the NSAID flurbiprofen, has been stopped after failure in a Phase III clinical trial [21]. The results of this multicenter, randomized, double-blind, placebo-controlled trial did not show any slowing of cognitive decline after 18 months of treatment with tarenflurbil. While the reasons for the clinical failure of tarenflurbil are unknown, low penetration across the blood–brain barrier (BBB) and, consequently, insufficient target engagement in the brain may be likely explanations [21].

The BBB separates the circulating blood from the central nervous system (CNS) and is comprised of endothelial cells, astrocytes and pericytes. Tight junctions between the endothelial cells are an essential part of the BBB because they close the intracellular space and limit the paracellular flux of hydrophilic molecules across the BBB. The brain endothelial cells express a large number of specialized transporters and receptors, including carriers for glucose and amino acids. Therefore, the BBB plays a crucial role in the regulation of the constancy of the internal environment of the brain and is essential for the supply of the CNS with nutrients. Furthermore, it protects the brain from the peripheral circulation and toxic substances and restricts the transport of many therapeutically important drugs from the blood into the brain, including Alzheimer drugs, anticancer drugs, antibiotics and a wide variety of CNS-active drugs [22-24].

Over the past few years, a number of different strategies have been devised to overcome the BBB such as osmotic opening of the tight junctions, the direct surgical administration of drugs into the brain or the development of drug carriers such as liposomes or nanoparticles [15,25-27]. However, the most notable and promising progression has been achieved by the use of nanotechnology. Liposomes as well as solid lipid nanoparticles or different polymeric nanoparticles have been successfully used for the transport of drugs across the BBB and into the brain [28]. Compared to free drug molecules or pro-drugs, the usage of nanoparticles possesses advantages such as a high drug-loading capacity of the nanoparticles. Furthermore, the drugs are protected against chemical or enzymatic degradation. In addition, nanoparticles can be actively targeted to a tissue via surface modifications of the nanoparticles [29].

In this study, we used polylactide (PLA) as the starting polymer for the nanoparticle preparation. By an emulsification-diffusion method, racemic flurbiprofen was embedded in the PLA nanoparticles. We decided to use flurbiprofen as a candidate substance since flurbiprofen has already been approved by the Food and Drug Administration (FDA) and is freely available as an over-the-counter medicine. We studied the transport of nanoparticulate flurbiprofen in an *in vitro* BBB model, and we could convincingly demonstrate that γ-secretase modulation *in vitro* was significantly enhanced after BBB penetration when flurbiprofen was delivered with nanoparticles compared to flurbiprofen alone.

## Methods

### Reagents and chemicals

Poly(L-lactide) (PLA, viscosity approximetely 1.0 dL/g), flurbiprofen and polyvinyl alcohol (PVA) were obtained from Sigma (Steinheim, Germany). Lumogen® F orange 240 was provided by BASF (Ludwigshafen, Germany). All other reagents were of analytical grade and used as received.

### Nanoparticle preparation

PLA nanoparticles were formed by an emulsification-diffusion technique. Briefly, 100 mg of PLA, 10 mg of flurbiprofen and 150 μg of Lumogen® orange were dissolved in 2 ml dichloromethane (DCM). For the control PLA nanoparticles, 100 mg of PLA and 150 μg of Lumogen® orange were dissolved in 2 ml DCM. For both formulations, the organic phase was added to 6 ml aqueous solution of PVA (2%, w/v). This mixture was homogenized in an ice bath for 30 minutes at 24,000 rpm (Ultra Turrax®, IKA, Staufen, Germany) and diluted with 6 ml PVA solution (1%, w/v). DCM was removed by stirring the emulsion over night at room temperature. Finally, the particles were collected by centrifugation at 20,000 *g* for 10 minutes (Eppendorf, Hamburg, Germany) and washed twice with purified water before lyophilization.

### Freeze-drying of the samples

For the lyophilization process a freeze-dryer Epsilon 1–4 (Martin Christ Gefriertrocknungsanlagen GmbH, Osterode am Harz, Germany) was used. Aliquots of the nanoparticles suspension (100 μl) were dispensed into 2 ml Lyovials and diluted with 100 μl trehalose solution (6%, w/v) as a cryoprotective agent. The freeze-drying cycle was performed according to an established protocol. First, the samples were frozen at -40°C for three hours. In the second step, primary drying was performed at a temperature of -34°C for 24 hours and a vacuum of 0.05 mbar, followed by a secondary drying phase for 11 hours at 20°C and a vacuum of 0.025 mbar. At the end of the drying process the vials were sealed and removed.

### Nanoparticle characterization

Nanoparticles were analyzed with regard to particle diameter and polydispersity by photon correlation spectroscopy (PCS) and zeta potential was measured by microelectrophoresis using a Malvern Zetasizer Nano ZS (Malvern Instruments Ltd., Malvern, UK). Prior to measurement the samples were diluted with purified water.

### Determination of flurbiprofen loading

The amount of flurbiprofen incorporated into the nanoparticles was determined by a HPLC method in which 1 mg nanoparticles was incubated in 1 ml acetonitrile for five minutes at room temperature. The sample was centrifuged (20,000 *g* for 10 minutes) and the chromatographic separation was carried out using aliquots of the supernatant. The aliquots (20 μl) were injected into a Phenomenex Gemini NX 250 × 4.6 mm, 5 μm particle, C18 column (Phenomenex, Aschaffenburg, Germany). The flow rate was set to 1 ml/minute during the separation, with the mobile phase composed of acetonitrile and 0.1% (v/v) trifluoroacetic acid (57.5: 42.5, v/v). The eluate was analyzed at a wavelength of 245 nm.

### *In vitro* release of flurbiprofen

For each point in time individual samples were prepared as follows: 1 mg nanoparticles were incubated in 1 ml phosphate buffer (0.1 mM, pH = 7.5) at 37°C under constant shaking. At defined points in time (0, 0.5, 1, 3, 5, 7, 24 hours) one sample was centrifuged (20,000 *g* for 10 minutes). The amount of the released drug was determined in the supernatant by HPLC as described above.

### Nanoparticles reconstitution

The freeze-dried nanoparticles were always reconstituted prior to the cell culture experiments. Therefore, 40 mg nanoparticles were dissolved in 1 ml purified water and vortexed for two minutes.

### Cell culture

The mouse brain endothelial cell line bEnd.3 (ATCC, Manassas, VA, USA) was cultured in DMEM (Gibco, Darmstadt, Germany) high glucose medium containing 10% fetal bovine serum and 100 U/ml penicillin/streptomycin (Gibco, Darmstadt, Germany). For the experiments, 5 × 10^4^ cells per cm^2^ were seeded and the experiments were performed after three days when the cells were post-confluent. APP751 overexpressing CHO cells (7WD10) were cultured in DMEM high glucose medium containing 10% fetal bovine serum, 1 mM sodium pyruvate (Gibco, Darmstadt, Germany), 100 U/ml penicillin/streptomycin and 400 μg/ml geneticin (Calbiochem, Nottingham, UK). For the experiments, 3 × 10^4^ cells per cm^2^ were seeded, and after 24 hours cells were either treated or co-cultured with the bEnd.3 in the *in vitro* BBB model.

### Measurement of cytotoxicity

The cytotoxicity of free flurbiprofen or PLA-flurbiprofen nanoparticles was assessed using the alamarBlue® reagent (Invitrogen, Karlsruhe, Germany). bEnd.3 cells were seeded on 96-well plates (Greiner, Frickenhausen, Germany) and after reaching post-confluency, cells were treated with increasing concentrations of free or nanoparticulate flurbiprofen, ranging from 25 μM to 750 μM (which corresponded to approximately 32 μg/cm^2^ to 942 μg/cm^2^ nanoparticles). The unit μg per cm^2^ refers to the amount of nanoparticles which are administered to the cells and this unit reflects possible local sedimentation on the surface of the cells, which locally might lead to different concentrations. After 72 hours, cells were incubated for another four hours with 1 × alamarBlue® in medium. The absorbance was measured with an Anthos plate reader 2010 (Anthos Labtec, Salzburg, Austria) using a 570 nm measurement filter and a 600 nm reference filter. The cell viability was calculated as percentage of absorbance in relation to vehicle control treated cells.

### Measurement of the transepithelial electrical resistance of endothelial cells

The transepithelial electrical resistance (TER) was used to analyze the toxicity of the nanoparticles for endothelial cells. bEnd.3 cells were seeded on 24-transwell cell culture inserts (ThinCerts™, Greiner Bio-One, Frickenhausen, Germany) and placed into the cellZscope® device [30]. The TER of the cells was measured automatically every hour under physiological conditions by impedance spectroscopy. When cells were post-confluent, equal amounts of drug-loaded and unloaded nanoparticles (approximately 2.4 mg nanoparticles per cm^2^) were added luminally and the TER was measured (this concentration corresponds to 750 μM nanoparticulate flurbiprofen).

### Cellular binding and uptake of nanoparticles

bEnd.3 cells were cultured in 24-well plates (Greiner, Frickenhausen, Germany) and treated with approximately 100 μg/cm^2^ PLA-flurbiprofen nanoparticles for four hours at 37°C. After the incubation, cells were washed twice with PBS (Invitrogen, Karlsruhe, Germany) and subsequently trypsinized and harvested. Fixing was performed with FACS-Fix (10 g/L paraformaldehyde (PFA) and 8.5 g/L NaCl in PBS, pH 7.4) before flow cytometry analysis. Per sample, 10^4^ cells were counted using FACSCalibur and CellQuest Pro software (Becton Dickinson, Heidelberg, Germany). The fluorescent labeling of the nanoparticles via Lumogen® F Orange 240 allowed a detection at 524/539 nm. To study the endocytotic uptake of the PLA nanoparticles, bEnd.3 cells were grown on glass coverslips (Marienfeld, Lauda-Königshofen, Germany) and treated with approximately 100 μg/cm^2^ PLA-flurbiprofen nanoparticles at 4°C or 37°C for one hour or four hours. After the incubation, cells were put on ice and washed with PBS pH2 to remove the surface-bound nanoparticles, mimicking the acidic environment of endosomes where ligands dissociate from their receptor after internalization [31]. Cells were fixed with 4% PFA and 0.12 M sucrose in PBS for 10 minutes at room temperature and the cell nuclei were stained with 2 μM DRAQ5™ (Biostatus Limited, Leicestershire, UK) for 10 minutes at room temperature. Samples were embedded in Prolong® Gold antifade reagent (Invitrogen, Darmstadt, Germany) and the confocal laser scanning microscope (CLSM) study was performed with a CLSM equipped with ZEN 2008 software (LSM 710; Zeiss, Jena, Germany).

### Treatment of 7WD10 with nanoparticles

To examine the biological activity of flurbiprofen-loaded nanoparticles, 7WD10 were treated with free or nanoparticulate flurbiprofen, ranging from 50 μM to 250 μM flurbiprofen. The administered concentration of the nanoparticles was adjusted to the free flurbiprofen, which corresponds to approximately 65 μg/cm^2^ to 317 μg/cm^2^ nanoparticles. After 48 hours, the supernatants were collected and centrifuged at 18,000 *g* for 20 minutes at 4°C. Levels of Aβ were measured by an Aβ specific ELISA.

### Transport assay of nanoparticulate flurbiprofen in an *in vitro* BBB model

bEnd.3 cells were seeded on 24-transwell cell culture inserts. After reaching post-confluency, bEnd.3 cells were co-cultured with 7WD10 in the lower compartment and bEnd.3 cells were treated with 300 μM free flurbiprofen or nanoparticulate flurbiprofen, ranging from 300 μM to 750 μM flurbiprofen, which corresponds to approximately 380 μg/cm^2^ to 942 μg/cm^2^ nanoparticles. After 72 hours, the supernatants of the lower compartment were collected and centrifuged at 18,000 *g* for 20 minutes at 4°C. Levels of Aβ were measured by an Aβ specific ELISA.

### Measurement of Aβ species by ELISA

The levels of Aβ_40_ and Aβ_42_ peptides were determined using a cell-based sandwich ELISA assay as described [32]. Briefly, the monoclonal antibody IC16 (1:250 in PBS, pH 7.2) raised against amino acids 1 to 15 of the Aβ sequence was used as a capture antibody. To generate standard curves, synthetic Aβ_40_ and Aβ_42_ peptides (JPT Peptide Technologies, Berlin, Germany) were used. These Aβ peptides were solubilized in dimethyl sulfoxide (DMSO) at 10 μg/ml and aliquots were stored at -80°C. The capture antibody was inclubated overnight in 96-well high-binding microtiter plates at 4°C. After the capture antibody was removed, conditioned media samples (20 μl for detection of Aβ_40_ and 100 μl for Aβ_42_) and freshly diluted Aβ peptide standards (125 to 6,000 pg/ml in PBS containing 0.05% Tween-20, 1% BSA) were added. Subsequently, C-terminal detection antibodies specific for Aβ_40_ and Aβ_42_ labeled with horseradish peroxidase (HRP) using the Pierce EZ-Link™ Plus Activated Peroxidase kit (Thermo Fisher Scientific, Rockford, IL, USA) were diluted in PBS containing 0.05% Tween-20, 1% BSA, added to each well, and incubated overnight at 4°C. Plates were washed three times with PBS containing 0.05% Tween-20 and once with PBS. Then, 50 μl of TMB (3,3',5,5'-Tetramethylbenzidin) ELISA Peroxidase Substrate (Interchim, Montlucon cedex, France) was added and incubated for 1 to 10 minutes at room temperature in the dark. The reaction was stopped by adding 50 μl of 2 M H_2_SO_4_ and the absorbance was measured using a Paradigm microplate reader (Beckman Coulter, Krefeld, Germany) at 450 nm. The levels of the Aβ_40_ and Aβ_42_ peptides were normalized to Aβ total (Aβ_40_ + Aβ_42_) and the average of triplicate measurements for each concentration was normalized to the control condition (DMSO or unloaded PLA nanoparticles).

### Nanoparticle plasma protein binding assay

To obtain human plasma, blood was taken at the ENT department at the Medical University Mainz from 15 different seemingly healthy donors in k_2_EDTA coated tubes (Greiner, Frickenhausen, Germany) to prevent blood clotting. The blood samples were labeled anonymously and could not be traced back to a specific donor. Studies were approved by the local ethics committee of the University Medical Center of the Johannes Gutenberg-University of Mainz, and informed consent was obtained in accordance with the Declaration of Helsinki. The PLA nanoparticles were incubated with equal amounts of human plasma for different time points (5, 15, 30 and 60 minutes), loaded onto a sucrose cushion (0.7 M in PBS) and centrifuged through the cushion to separate nanoparticle-protein complexes from plasma (12,000 rpm for 20 minutes at 4°C). Pellets were washed three times with PBS and proteins were eluted from the recovered particles by adding an equal volume of SDS sample buffer (62.5 mM Tris–HCl pH 6.8; 2% (w/v) SDS, 10% glycerol, 50 mM dithiothreitol (DTT), 0.01% (w/v) bromophenol blue) to the pellet and incubated at 95°C for five minutes. Proteins were separated on a 12% SDS-polyacrylamide gel. To visualize the kinetic evolution of the protein corona, the SDS-polyacrylamide gel was stained with Coomassie brilliant blue R-250 (Bio-Rad, München, Germany) and protein quantification was performed using the BioRad Protein Assay (Bio-Rad, München, Germany). To examine the presence of apolipoproteins in the nanoparticle-protein complex, proteins were transferred onto a polyvinylidene difluoride (PVDF) membrane. Membranes were blocked with 5% non-fat dry milk in Tris-buffered saline (TBS) containing 0.01% Tween-20 and the following antibodies were used: α-apoA4 (Cell Signaling, Boston, MA, USA ); α-apoE and rabbit-α-mouse immunoglobulin G (IgG) antibody conjugated with HRP (Santa Cruz, Dallas, TX, USA).

### Statistical analysis

All graphs and statistics were performed using the GraphPad Prism 4 software (GraphPad, La Jolla, CA, USA). Data were analyzed by two-way analysis of variance (ANOVA) coupled to Bonferroni post-tests for multiple comparisons. *P* <0.05 was considered as statistically significance.

## Results

### Preparation and characterization of PLA nanoparticles

To investigate a novel approach to deliver potential drugs against AD into the brain, we established an *in vitro* BBB model to analyze the transport and functionality of the Aβ_42_ lowering drug flurbiprofen embedded in PLA nanoparticles. The PLA-flurbiprofen nanoparticles were prepared by an emulsification-diffusion method. For the visualization of the nanoparticles, Lumogen® F Orange 240 (kindly provided by BASF) was added during the preparation. The particles had a size between 213.6 and 218.1 nm. The amount of incorporated flurbiprofen was determined by HPLC (Table 1).

**Table 1:** Physicochemical characteristics of polylactide (PLA) nanoparticles

**Formulation**	**Mean particle diameter (nm)**	**Polydispersity index**	**Zeta potential (mV)**	**Flurbiprofen loading (μg/mg)**
PLA-flurbiprofen nanoparticles	218.1	0.053	-17.4	60.2
PLA nanoparticles	213.6	0.048	-27.6	-

### Determination of cell toxicity

**Figure 1:**
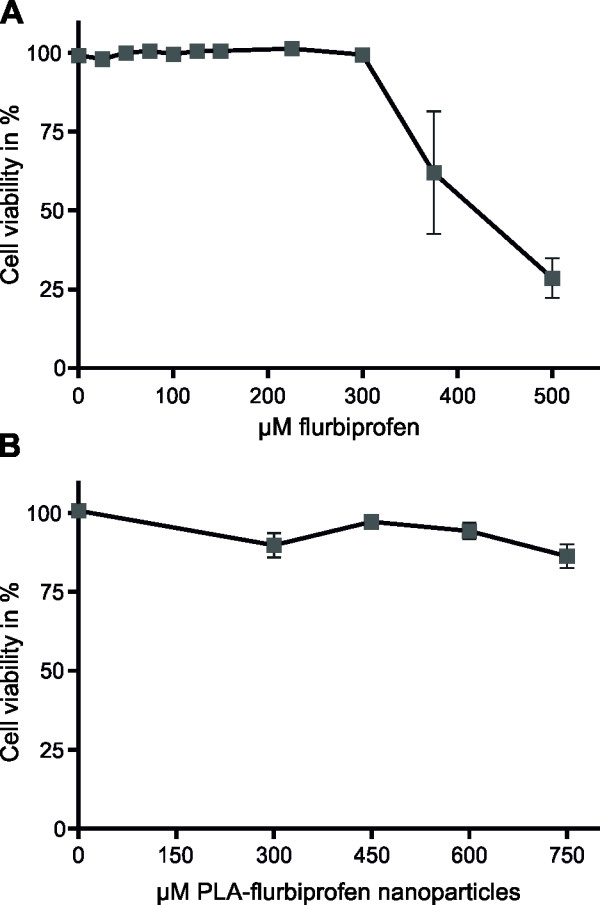
**Cytotoxicity of free or nanoparticulate flurbiprofen.** bEnd.3 cells were treated with the indicated concentrations of free flurbiprofen **(A)** or PLA-flurbiprofen nanoparticles **(B)** for 72 hours. The cytotoxicity was assessed after a four hour incubation with 1 × alamarBlue®. The absorbance was measured and the cell viability was calculated as a percentage of absorbance in relation to vehicle control treated cells. The data represent mean ± SEM of n ≥3. PLA, polylactide; SEM, standard error of the mean.

**Figure 2:**
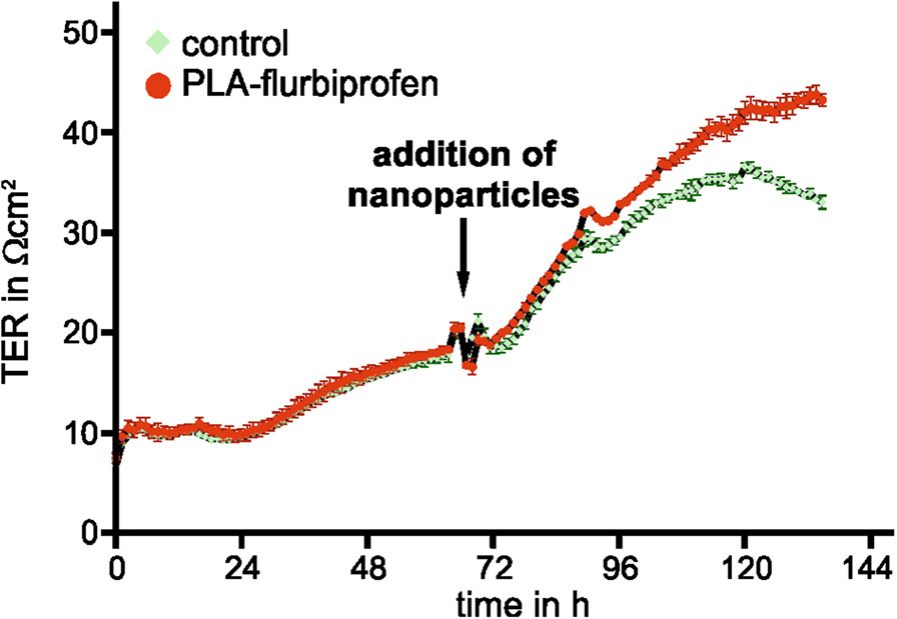
**Influence of PLA-flurbiprofen nanoparticles on the integrity of the endothelial cell barrier.** bEnd.3 cells were cultivated on cell culture inserts in a cellZscope® device. When cells were post-confluent, approximately 2.4 mg nanoparticles per cm^2^ (corresponding to 750 μM nanoparticulate flurbiprofen) were added to the luminal side of the endothelial cells and the TER was measured every hour by impedance spectroscopy. PLA, polylactide; TER, transepithelial electrical resistance.

The cytotoxicity of the PLA-flurbiprofen nanoparticles was assessed using the alamarBlue® cell viability reagent. Therefore, post-confluent bEnd.3 cells were incubated with increasing concentrations of free flurbiprofen or PLA-flurbiprofen nanoparticles. As reported earlier by us and others, free flurbiprofen has a cytotoxic potential at concentrations above 300 μM (Figure 1A) [19,33]. In contrast, nanoparticulate flurbiprofen showed no cytotoxic potential (Figure 1B). The toxicity of the nanoparticles was further analyzed by the measurement of their influence on the integrity of endothelial cells (Figure 2). Therefore, the TER of the cells was measured by impedance spectroscopy using a cellZscope® device [30]. bEnd.3 were cultured on cell culture inserts and after reaching post-confluency, cells were treated with 750 μM nanoparticulate flurbiprofen. The nanoparticles showed no interference with the TER development, even at this high concentration. Taken together, the cell viability as well as the integrity of the endothelial cells was not impaired by the nanoparticulate flurbiprofen and, therefore, any cytotoxic effect could be excluded.

### Biological activity of nanoparticles

**Figure 3:**
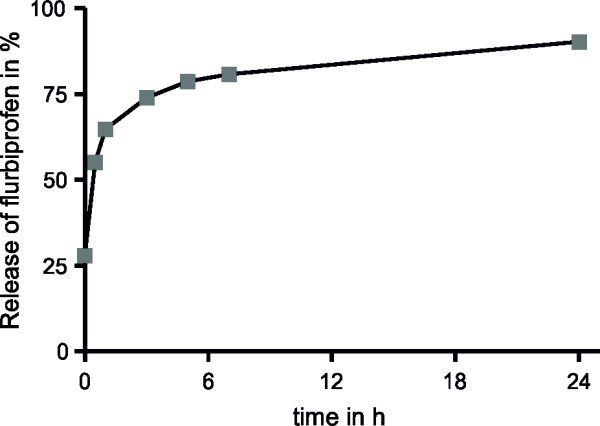
**Flurbiprofen is released over time from the PLA nanoparticles.** PLA-flurbiprofen nanoparticles were dissolved in an aqueous solution at pH 7.5 and the amount of flurbiprofen in the solution was measured by HPLC at different time points. The release is shown as the percentage of the total amount of incorporated flurbiprofen in the nanoparticles. PLA, polylactide.

**Figure 4:**
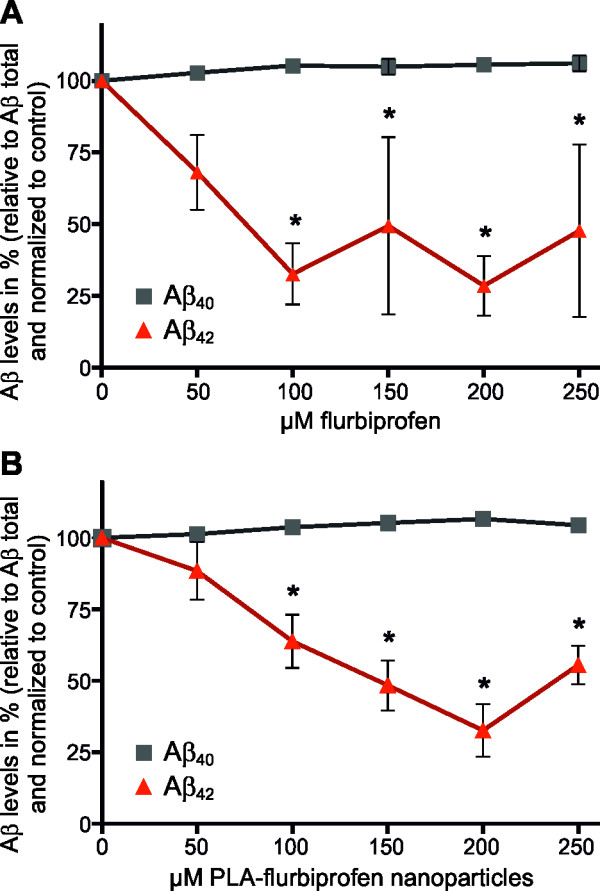
**Aβ generation in cultured cells treated with free or nanoparticulate flurbiprofen.** 7WD10 cells were treated with the indicated concentrations of free flurbiprofen **(A)** or PLA-flurbiprofen nanoparticles **(B)** for 48 hours. Levels of Aβ were measured by an Aβ specific ELISA. The data represent mean ± SEM of three independent experiments. * Statistically significant difference (*P* <0.05, two-way ANOVA) between Aβ levels of vehicle-treated and drug-treated cells are indicated. Aβ, amyloid-β; ANOVA, analysis of variance; PLA, polylactide; SEM, standard error of the mean.

Although we could convincingly demonstrate the tolerance of endothelial cells to nanoparticulate flurbiprofen, we had to establish whether nanoparticulate flurbiprofen had any biological activity on modulating the γ-secretase function. Therefore, we first analyzed the *in vitro* release of flurbiprofen from the PLA nanoparticles at a physiological pH by incubating 1 mg nanoparticles in 1 ml phosphate buffer (pH = 7.5) at 37°C. By HPLC, we observed that flurbiprofen is constantly released from the nanoparticles over time with an initial rapid release followed by a slower exponential phase (Figure 3). Consequently, we analyzed the influence of nanoparticulate flurbiprofen on γ-secretase activity using APP overexpressing cells (7WD10). The 7WD10 cells were incubated with free flurbiprofen or PLA-flurbiprofen nanoparticles (both ranging from 50 μM to 250 μM). After 48 hours, tissue culture supernatants were collected and the amounts of Aβ_40_ and Aβ_42_ were measured by an Aβ specific ELISA. As reported earlier by us and others, free flurbiprofen specifically modulates γ-secretase activity by decreasing the levels of Aβ_42_ in a concentration dependent manner whereas the levels of Aβ_40_ remained unaffected (Figure 4A) [17,19]. For the treatment with the PLA-flurbiprofen nanoparticles, the administered concentration of the nanoparticles was adjusted to the free flurbiprofen to compare the effects of the nanoparticulate and the free drug. The nanoparticulate flurbiprofen reduced the amount of the Aβ_42_ peptides in a concentration dependent manner (Figure 4B). The levels of Aβ_40_ remained unaffected. The decrease of the Aβ_42_ levels by the PLA-flurbiprofen nanoparticles is achieved by the *in vitro* release of the drug from the nanoparticles, as shown in Figure 3. Thus, the nanoparticulate flurbiprofen exhibited a biological activity to modulate γ-secretase *in vitro* comparable to free flurbiprofen.

### Cellular binding and uptake of nanoparticles

**Figure 5:**
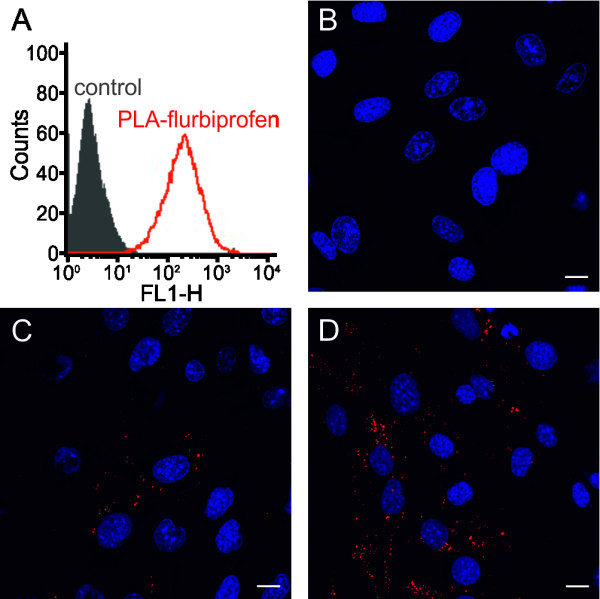
**Cellular binding of the PLA-flurbiprofen nanoparticles. (A)** bEnd.3 cells were treated with approximately 100 μg/cm^2^ PLA-flurbiprofen nanoparticles for four hours at 37°C and the cellular binding was quantified by flow cytometry. The data are shown as histograms of the FL1-H channel (red: PLA-flurbiprofen nanoparticles, black: untreated control). **(B-D)** bEnd.3 cells were treated with approximately 100 μg/cm^2^ nanoparticles. To inhibit endocytosis, the cells were treated at 4°C **(B)**. For the cellular uptake, cells were treated for one hour **(C)** or four hours **(D)** at 37°C. After the incubation period, the cells were put on ice and washed with acidic PBS to remove the surface-bound nanoparticles. The cells were fixed with paraformaldehyde and cell nuclei were stained with DRAQ5^TM^. Scale bar, 10 μm. PLA, polylactide.

To analyze the potential of nanoparticulate flurbiprofen to cross a cellular monolayer, we first examined the cellular binding and uptake of the PLA-flurbiprofen nanoparticles by endothelial cells. Therefore, bEnd.3 cells were incubated with the nanoparticles for four hours at 37°C and subsequently analyzed by flow cytometry (Figure 5A). Compared to the untreated control, cellular binding of the PLA-flurbiprofen nanoparticles was detected. The cellular uptake of the nanoparticles was further studied by CLSM. Post-confluent bEnd.3 cells were incubated with nanoparticles for one to four hours at either 37°C or at 4°C as a control to inhibit endocytosis. At 4°C, the bEnd.3 cells exhibited no signal of the nanoparticles demonstrating that the nanoparticles were not endocytosed (Figure 5B). However, at 37°C, the nanoparticles were endocytosed by the bEnd.3 cells (Figure 5C) and this uptake increased with longer incubation time (Figure 5D).

### Transport of nanoparticles across endothelial cells

**Figure 6:**
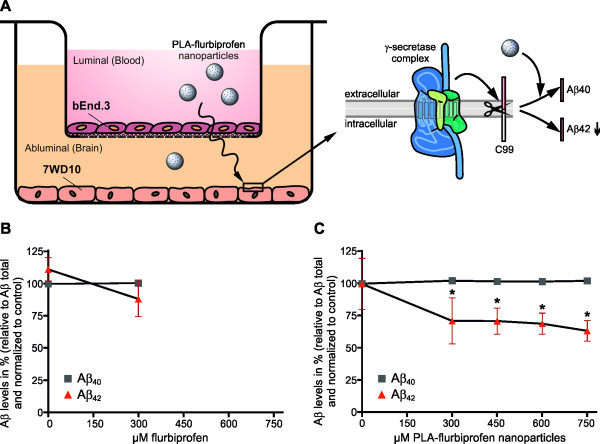
**Influence of free or nanoparticulate flurbiprofen on Aβ generation in an *in vitro *BBB model.** 7WD10 cells were co-cultured abluminal with post-confluent bEnd.3 cells in the luminal compartment **(A)**. bEnd.3 cells were treated with the indicated concentrations of free flurbiprofen **(B)** or PLA-flurbiprofen nanoparticles **(C)** for 72 hours. Levels of Aβ in the abluminal compartments were measured by an Aβ specific ELISA. The data represent mean ± SEM of three independent experiments. * Statistically significant difference (*P* <0.05, two-way ANOVA) between Aβ levels of vehicle-treated and drug-treated cells are indicated. Aβ, amyloid-β; ANOVA, analysis of variance; BBB, blood-brain barrier; PLA, polylactide; SEM, standard error of the mean.

To study the ability of PLA nanoparticles to transport flurbiprofen across an *in vitro* BBB model, bEnd.3 cells were cultivated in the luminal compartment of cell culture inserts until a post-confluent monolayer had grown (Figure 6A). Then, 7WD10 were co-cultured abluminally together with the bEnd.3 cells. After confirmation of the tightness of the endothelial cell monolayer by TER measurement, nanoparticles were added to the bEnd.3 cells at the luminal side. As a readout for the transport of nanoparticulate NSAIDs, we collected the medium in the abluminal compartment after 72 hours and measured the γ-secretase activity by determining the levels of Aβ_40_ and Aβ_42_ using a specific ELISA. Furthermore, we measured the amount of flurbiprofen by HPLC. Luminally administered free flurbiprofen (300 μM) had no effect on the levels of Aβ_40_ and Aβ_42_ in the abluminal compartment (Figure 6B). We only administered 300 μM free flurbiprofen in this experimental setup due to its cytotoxic potential at higher concentrations (Figure 2A). By HPLC, we measured the concentration of flurbiprofen in the abluminal compartment and we could detect that approximately 10% of the initial concentration was diffusing across the endothelial cell monolayer, which was not sufficient to reduce Aβ_42_ (Table 2). When flurbiprofen-loaded nanoparticles were added luminally to the endothelial cells, Aβ_42_ levels decreased in a concentration dependent manner whereas the levels of Aβ_40_ remained unchanged (Figure 6C). Since the IC_50_ for flurbiprofen induced γ-secretase modulation is about 150 to 200 μM, we hypothesize that the final concentration of flurbiprofen in the abluminal compartment, most likely still bound to nanoparticle components, is therefore sufficient to reduce the Aβ_42_ levels. These results strongly indicate that the nanoparticles were transported in this *in vitro* BBB model and that the embedded flurbiprofen was able to reduce Aβ_42_ levels by modulating the γ-secretase activity.

**Table 2:** Concentration of flurbiprofen in the abluminal compartment measured by HPLC

**μM luminally administered**	**μM abluminally measured by HPLC**	**% of administered concentration**
300 μM flurbiprofen	41.85	13.95
300 μM PLA-flurbiprofen nanoparticles	31.56	10.52
450 μM PLA-flurbiprofen nanoparticles	46.82	10.40
600 μM PLA-flurbiprofen nanoparticles	63.95	10.66
750 μM PLA-flurbiprofen nanoparticles	83.70	11.16

### Human blood plasma corona of the nanoparticles

**Figure 7:**
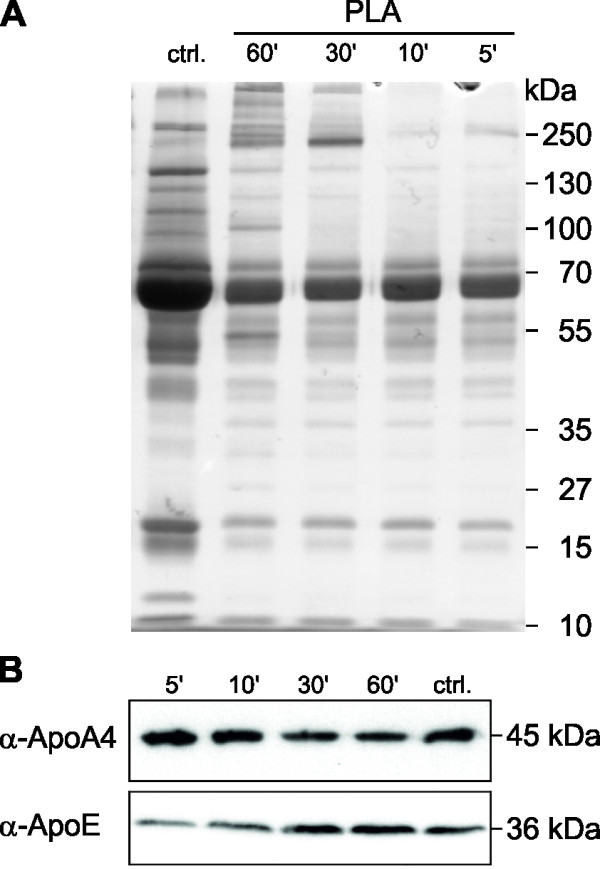
**ApoE and ApoA4 are part of the protein corona of the PLA nanoparticles.** PLA nanoparticles were incubated with equal amounts of human plasma for different time points and the nanoparticles-protein complexes were analyzed by SDS-PAGE **(A)** and Western blot **(B)**. Human blood plasma served as control (ctrl.). Apo, apolipoprotein; PLA, polylactide.

When nanoparticles enter a biological fluid, proteins rapidly compete for binding to the nanoparticle surface, leading to the formation of a protein corona that critically defines the biological identity of the particle. The properties of such a particle-protein complex differ significantly from those of the formulated particle. Hence, the further biological responses of the body as well as the biodistribution of the nanoparticles are expected to be influenced by the nanoparticle-protein complex [34]. Therefore, the PLA nanoparticles were incubated with human blood plasma for several time points and we examined the binding of plasma proteins to the nanoparticles. The protein binding profiles were visualized by SDS-PAGE, demonstrating that an exposure for five minutes was already sufficient for an efficient corona formation (Figure 7A). Also, the amount of corona proteins increased over time, albeit the corona seems to change only quantitatively rather than qualitatively. Furthermore, we examined the presence of apolipoproteins in the protein corona (Figure 7B). Apolipoproteins are part of the circulating lipoproteins and serve as receptor ligands for lipid and cholesterol uptake and metabolism, and it was suggested that apolipoprotein-modified nanoparticles are able to transport drugs across the BBB [35-38]. We observed that apolipoprotein E (ApoE) and apolipoprotein A4 (ApoA4) are already present in the nanoparticle-protein complex after five minutes of exposure, and that the amount of ApoA4 slightly decreased and the ApoE concentrations significantly increased over time. The formation of the apolipoprotein corona on the PLA nanoparticles indicates a potential transport route of the nanoparticles through a lipoprotein receptor transcytosis pathway [37,39].

## Discussion

AD is a severe neurodegenerative disease affecting more than 35 million people worldwide, and is characterized by memory impairment, neurofibrillary tangles formation and extracellular accumulation of Aβ_42_ in insoluble plaques. The Aβ_42_ peptide is generated through the proteolytic processing of APP, and its accumulation or increased generation is thought to be one of the primary events in the pathogenesis of AD [12-14,40]. According to this hypothesis, reduced generation or enhanced clearance of Aβ_42_ peptides and plaques would be expected to modify the disease course in AD [41]. Recently, the Aβ_42_ lowering agent tarenflurbil, the *R*-enantiomer of flurbiprofen, failed in a phase III clinical trial, presumably because it could not cross the BBB in high enough concentrations [21]. Generally, brain penetration of CNS active drugs is mostly limited by the BBB, and several strategies have been devised to overcome this barrier to deliver therapeutic drugs to the brain [42]. To date, drug carriers, such as nanoparticles, have been studied intensively for neurological disorders, cancer and other diseases, and some of them are in different trial phases or even commercially available [43,44]. In this study, we examined the efficiency of nanoparticulate flurbiprofen to cross the BBB in order to decrease selectively the levels of Aβ_42_ peptides in the brain.

We decided to study PLA-nanoparticle formulations because this starting material is FDA-approved, biodegradable and biocompatible [29,45]. In addition, we decided to use flurbiprofen as a GSM because it is also FDA-approved, commercially available and, most importantly, its GSM activity has been studied intensively *in vivo* and *in vitro*[17,19]. First, the cytotoxic potential of the PLA-flurbiprofen nanoparticles in comparison to free flurbiprofen was investigated. Compared to free flurbiprofen, the PLA-flurbiprofen nanoparticles did not affect cell viability. Even if high amounts of nanoparticles were administered to the endothelial cells, and including that the nanoparticles may sediment in the culture medium on the surface of the cells which may lead to different local concentrations, these amounts did not perturb the integrity of an endothelial barrier. Thus, the embedding of flurbiprofen in nanoparticles disguised its cytotoxic potential which enables the application of higher concentrations on endothelial cells without any cytotoxic potential. The cellular binding and uptake of the nanoparticles was observed by flow cytometry and CLSM. These data confirmed the cellular binding as well as a time-dependent uptake of the nanoparticles by endothelial cells. Next, the *in vitro* release of flurbiprofen from the nanoparticles was investigated, which showed that the drug is constantly released over the time. Consequently, we examined the biological activity of the nanoparticulate flurbiprofen compared to free flurbiprofen in the APP overexpressing cell line 7WD10. Free flurbiprofen specifically decreased Aβ_42_ levels with a maximal effect size of approximately 70%, in good agreement with previous studies [17,19]. For the PLA-flurbiprofen nanoparticles, a similar decrease in Aβ_42_ levels was detected. Since flurbiprofen is released constantly over time from the PLA nanoparticles, its biological activity to modulate γ-secretase activity *in vitro* was comparable to free flurbiprofen. Based on these observations that the PLA-flurbiprofen nanoparticles were able to lower Aβ_42_ levels and were endocytosed by endothelial cells, we studied their transport in an *in vitro* BBB model. In these experiments, the endothelial cells were cultured luminally on cell culture inserts (representing the blood side) with the 7WD10 cell line in the abluminal compartment (representing the brain side). In this experimental setup, free flurbiprofen did not reduce Aβ_42_ levels, because it was not sufficiently transported across the endothelial cell monolayer at high enough concentration. This is in agreement with phase I dosing studies of *R*-flurbiprofen in humans [46]. In these studies, healthy volunteers were treated for 21 days with up to 800 mg twice daily of *R*-flurbiprofen. Maximal plasma concentrations of *R*-flurbiprofen up to 185 μM were measured, well in the range of the Aβ_42_-lowering activity of flurbiprofen in tissue culture experiments. However, flurbiprofen concentrations in the cerebrospinal fluid (CSF) of the volunteers were found to be more than 100-fold lower with an average CSF to plasma ratio of 0.5%. No significant changes in CSF Aβ_42_ levels were observed in *R*-flurbiprofen treated individuals compared to placebo controls. In the failed Phase III clinical study of *R*-flurbiprofen, CSF drug concentration were not measured but the highest dose administered in the trial was identical to the phase I study, predicting similarly low brain concentrations [21]. Importantly, we could detect a significant decrease of Aβ_42_ levels for the nanoparticulate flurbiprofen. In earlier studies, we were able to demonstrate that drug-loaded nanoparticles exhibited a higher biological activity than the free drug alone [47]. The nanoparticulate drug had a lower IC_50_ value than the free drug molecule resulting in a higher efficiency of the nanoparticulate formulation. Furthermore, the nanoparticles may gradually sediment in the culture medium on the surface of the cells, which can locally lead to higher concentrations of the nanoparticulate flurbiprofen compared to free flurbiprofen. For the HPLC measurements, the total capacity of the abluminal compartment was used and the total concentration of free or nanoparticulate flurbiprofen was measured. Thus, total concentration of the nanoparticulate flurbiprofen may not be higher compared to free flurbiprofen, but if the sedimentation of the nanoparticles is taken into account, the concentrations of the nanoparticulate flurbiprofen can be locally higher resulting in significantly decreased levels of Aβ_42_. The embedding of flurbiprofen into nanoparticles not only disguises its cytotoxic potential, it further enables the administration of higher drug concentrations resulting in a sufficient transport of the drug across the endothelial cell monolayer. These data provide a proof of principle showing that even NSAIDs with low GSM activity can efficiently lower Aβ_42_ levels if transported sufficiently across the BBB. As a potential transport mechanism we would like to highlight the presence of the lipoproteins ApoA4 and ApoE in the protein corona of the nanoparticles. In biological fluids, proteins and other biomolecules bind to the surface of the nanoparticles and build a corona which determines their biological properties. For instance, members of the apolipoprotein family enable the endocytotic uptake through lipoprotein receptors. In a recent study, we were able to show that ApoE-modified nanoparticles were actively endocytosed by endothelial cells and that the low density lipoprotein receptor-related protein 1 (LRP1) is involved in this process [37]. Thus, the transport of the nanoparticulate flurbiprofen may be facilitated by the surface-bound proteins mimicking endogenous lipoproteins via endocytosis by transporters such as LRP1.

## Conclusions

Over the past years, the application of nanotechnology-based strategies for the treatment or diagnosis of AD has been investigated by many groups [43,48]. Some approaches, like this study, focused on the encapsulation of molecules into nanoparticles for delivery to the brain; others dealt with a reduction of amyloid plaques toxicity or focused on the early detection of the disease [49]. In this study, we could show that after embedding in polymeric nanoparticles, flurbiprofen can be transported across the BBB and retains its biological activity. However, some improvements and optimizations of the nanoparticles are required for future applications. The use of other GSMs with higher potency, or surface modifications such as coupling the nanoparticles with peptides or ligands, which can achieve a higher bioavailability or a specific targeting to the brain, are just some examples for achievable improvements [15,20]. To date, an enormous amount of work has been invested in the sophisticated surface-functionalization of drug carriers to improve their targeting and/or bioactivity, but the potential impact of the protein corona on the success or failure of these strategies has been mostly neglected so far [34,50,51]. However, we show for the first time that the blood plasma protein corona on PLA nanoparticles is established rapidly, is complex and appears to change predominantly only quantitatively over time. The surface-functionalization of nanoparticles with apolipoproteins has been shown to facilitate translocation through the BBB [37,38,52], and we assume that the observed ‘natural functionalization’ of the PLA nanoparticles with ApoE and ApoA4 may facilitate the BBB transport of the nanoparticulate flurbiprofen via endocytosis using transporters such as LRP1 [37]. Whether further surface modifications may increase BBB transport remains to be investigated. The surface-functionalization of drug carriers is labor and cost-intensive and therefore often limited to (pre)clinical studies in nanobiomedicine. Since the protein corona is established in physiological environments, this ‘natural biofunctionalization’ should be exploited to improve the bioactivity of drug carriers to overcome these limitations.

Taken together, we were able to show that the modification of usually nonpermeable drugs via embedding in nanoparticles results in an efficient transport across an endothelial cell monolayer and that these nanotechnology-based strategies are very promising to generate novel therapeutic options for AD and other CNS diseases.

## Abbreviations

AD: Alzheimer’s disease; ApoA4: Apolipoprotein A4; ApoE: Apolipoprotein E; APP: Amyloid precursor protein; Aβ: Amyloid-β; BBB: Blood–brain barrier; BSA: Bovine serum albumin; CHO: Chinese hamster ovary; CLSM: Confocal laser scanning microscopy; CNS: Central nervous system; COX: Cyclooxygenase; CSF: Cerebrospinal fluid; DCM: Dichloromethane; DMEM: Dulbecco’s modified Eagle’s medium; DMSO: Dimethyl sulfoxide; ELISA: Enzyme-linked immunosorbent assay; FDA: Food and Drug Administration; GSM: γ-secretase modulators; HPLC: High performance liquid chromatography; HRP: Horseradish peroxidase; NSAIDs: Nonsteroidal anti-inflammatory drugs; PBS: Phosphate-buffered saline; PFA: Paraformaldehyde; PLA: Polylactide; PVA: Polyvinyl alcohol; TER: Transepithelial electrical resistance.

## Competing interests

The authors declare that they have no competing interests.

## Authors’ contributions

SM designed the studies and wrote the manuscript. IZ, JS, DD and SB performed experiments. SW and SyW contributed to the experimental design and the writing of the manuscript. RHS, MD, RS, SR, MW, KL and HB contributed to the writing of the manuscript. CUP supervised the experimental design and entire work of the manuscript. All authors read and approved the final manuscript.
